# Scaling-Up eConsult: Promising Strategies to Address Enabling Factors in Four Jurisdictions in Canada

**DOI:** 10.34172/ijhpm.2023.7203

**Published:** 2023-09-05

**Authors:** Mylaine Breton, Catherine Lamoureux-Lamarche, Mélanie Ann Smithman, Erin Keely, Maxine Dumas Pilon, Alexander Singer, Gerard Farrell, Paula Louise Bush, Catherine Hudon, Lynn Cooper, Véronique Nabelsi, Élizabeth Côté-Boileau, Justin Gagnon, Isabelle Gaboury, Carolyn Steele Gray, Marie-Pierre Gagnon, Regina Visca, Clare Liddy

**Affiliations:** ^1^Centre de recherche Charles-Le Moyne, Université de Sherbrooke, Longueuil, QC, Canada; ^2^Department of Medicine, University of Ottawa, Division of Endocrinology/Metabolism, The Ottawa Hospital, Ottawa, ON, Canada; ^3^Collège québécois des médecins de famille, Family Medicine Center, St-Mary’s Hospital, McGill University, Montréal, QC, Canada; ^4^Department of Family Medicine, University of Manitoba, Winnipeg, MN, Canada; ^5^Department of Family Medicine, Memorial University, St. John, NL, Canada; ^6^Department of Family Medicine, McGill University, Montréal, QC, Canada; ^7^Centre de recherche du CHUS, Université de Sherbrooke, Sherbrooke, QC, Canada; ^8^Canadian Injured Workers Alliance, Thunder Bay, ON, Canada; ^9^Département des sciences administratives, Université du Québec en Outaouais, Gatineau, QC, Canada; ^10^Bridgepoint Collaboratory for Research and Innovation, Lunenfeld-Tanenbaum, Research Institute, Sinai Health System, University of Toronto, Institute of Health Policy, Management and Evaluation, Toronto, ON, Canada; ^11^Faculté des sciences infirmières, Université Laval, Québec City, QC, Canada; ^12^Department of Family Medicine, University of Ottawa, Ottawa, ON, Canada; ^13^C.T. Lamont Primary Health Care Research Center, Bruyère Research Institute, Ottawa, ON, Canada

**Keywords:** Primary Care, Scaling-Up, eConsult, Policy, Canada, Digital Health

## Abstract

**Background:** Effective healthcare innovations are often not scaled up beyond their initial local context. Lack of practical knowledge on how to move from local innovations to large-system improvement hinders innovation and learning capacity in health systems. Studying scale-up processes can lead to a better understanding of how to facilitate the scale-up of interventions. eConsult is a digital health innovation that aims to connect primary care professionals with specialists through an asynchronous electronic consultation. The recent implementation of eConsult in the public health systems of four Canadian jurisdictions provides a unique opportunity to identify different enabling strategies and related factors that promote the scaling up of eConsult across jurisdictions.

**Methods:** We conducted a narrative case study in four Canadian provinces, Quebec, Ontario, Manitoba, and Newfoundland & Labrador, over a 3-year period (2018–2021). We observed provincial eConsult committee meetings (n=65) and national eConsult forums (n=3), and we reviewed internal documents (n=93). We conducted semi-structured interviews with key actors in each jurisdiction (eg, researchers, primary care professionals, specialists, policy-makers, and patient partners) (n=40). We conducted thematic analysis guided by the literature on factors and strategies used to scale up innovations.

**Results:** We identified a total of 31 strategies related to six key enabling factors to scaling up eConsult, including: (1) multi-actor engagement; (2) relative advantage; (3) knowledge transfer; (4) strong evidence base; (5) physician leadership; and (6) resource acquisition (eg, human, material, and financial resources). More commonly used strategies, such as leveraging research infrastructure and bringing together various actors, were used to address multiple enabling factors.

**Conclusion:** Actors used various strategies to scale up eConsult within their respective contexts, and these helped address six key factors that seemed to be essential to the scale-up of eConsult.

## Background

**Key Messages**

**Implications for policy makers**

- Unlike previous work on scale-up in healthcare, this study makes an empirical contribution by identifying practical scale-up strategies used to address key enabling factors.
- A better understanding of these strategies is crucial to advance our ability to scale up promising health innovations and suggest concrete actions that actors can take to support scale-up efforts.
- Many identified strategies involve leveraging research infrastructure and bringing together various actors.

**Implications for the public**

This study identified strategies used in four Canadian provinces to support the expansion of eConsult. eConsult is a platform that allows primary care professionals to asynchronously consult specialists regarding their patients’ medical problems. It has been demonstrated to effectively improve access, patient satisfaction, and equity and reduce system costs. Expanding promising healthcare innovations by learning from strategies that supported the scale-up of eConsult will help improve care for the population.

The challenge of effectively scaling up promising innovations beyond pilot projects often hinders efforts to improve healthcare.^1-4^ A healthcare innovation is a new product, intervention, care pathway, or service that significantly benefits patients and health systems.^5-8^ Scaling up innovations involves iterative decisions, events, and actions to tackle infrastructure issues that arise across health system levels while expanding innovations to full scale to benefit targeted populations.^1,4,9^

 Canada faces persistent difficulties in scaling-up healthcare innovations,^1,2,10-14^ leading to its reputation as the “country of perpetual pilot projects.”^11^ Scaling up healthcare innovations in Canada presents a complex and multifaceted challenge that remains poorly understood. Health system leaders and experts have identified several potential barriers, including resources allocated primarily towards pilot projects rather than expansion, inadequate support for clinician-innovators, limited engagement of diverse actors including patients, a risk-averse culture, opaque decision-making processes, insufficient data infrastructure and access, fragmented health systems, ineffective change management, and a general lack of knowledge regarding the most effective strategies for scaling up innovations.^11,13,14^

 A growing body of evidence on the science of scaling up healthcare innovations has identified several enabling factors.^1,4,9,10,15-17^ These factors include (1) identifying population needs and system problems; (2) dedicating human, financial, and technical resources; (3) creating relational and political connections and actively involving key actors; (4) strong health professional leadership; (5) adapting to the political context and integrating with existing services; and (6) measuring and disseminating innovation and scale-up outcomes.

 However, two significant gaps persist in the current literature on scaling up healthcare innovations. Firstly, most studies focus on low- and middle-income countries, which provide valuable information but may differ in terms of resources, policies, governance, and infrastructure compared to high-income contexts.^1,4,17-19^ Secondly, there is a notable dearth of evidence on the operationalization of scale-up and strategies that key actors can adopt to address enabling factors.^20^ Thus, there is a need to study empirical scale-up processes and strategies within the context of high-income countries. Such evidence may provide much-needed guidance on expanding proven healthcare innovations to improve equitable health outcomes in universal health systems.

 eConsult is one of the most promising health innovations piloted in Canada to improve access to specialist care.^21-24^ eConsult is an asynchronous electronic communication platform used to transmit medical advice between a primary care professional and specialists in fields such as dermatology, cardiology, pediatrics, oncology, psychiatry, and geriatrics.^21-24^ Studies have demonstrated that eConsult reduces wait times, avoids face-to-face referrals, is associated with positive patient and professional experiences, reduces health system costs, and improves equity.^21-23^

**Figure 1:**
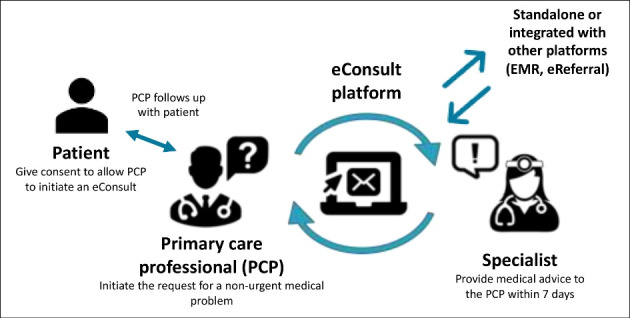
The eConsult Innovation Implemented in Four Canadian Provinces. Abbreviations: EMR, electronic medical record; PCP, primary care professionals.

 The Building Access to Specialists through eConsultation (BASE^TM^) eConsult model was developed and piloted in one region of the Canadian province of Ontario in 2009,^24^ then spread throughout Ontario and further expanded to other Canadian provinces. This evidence-based innovation began as a small proof-of-concept pilot project that was spread and is being scaled up in other Canadian provinces. eConsult is trademarked by the founders of BASE^TM^ eConsult, who charge minimal fees for service maintenance but do not charge for its use by other provinces. eConsult is free-of-charge for physicians and professionals. Specialists received a pro-rated fee for each eConsultation as part of a research project. Figure 1 illustrates the eConsult innovation implemented in the four provinces under study. The fact that the four provinces are at different stages of the scale-up process offers a unique learning opportunity.^25^

 Our study aims to identify the key factors that enabled the scale-up of eConsult and the strategies used to address them in four provinces in Canada. As part of this study, we define the *factors* enabling scale-up as the key areas identified to facilitate scale-up, whereas the *strategies* for scale-up are the actions taken by actors to address these factors.

## Methods

###  Design 

 The research used a narrative case study design.^26^ This design involved an in-depth examination of eConsult implementation by triangulating data from multiple sources and contexts and reconstructing each studied case in narrative form.^27^ The study included four Canadian provinces where eConsult scale-up was underway^28^: Quebec, Ontario, Manitoba, and Newfoundland & Labrador. In Canada, the health care system is publicly funded, but provinces are responsible for planning, administering, and delivering health care services, resulting in provincial differences in laws, regulations, and structures. The provinces under study had participated in a national Partnership for Health System Improvement grant and in the Canadian Foundation for Health Improvement’s (CFHI’s) Connected Medicine Initiative (2017–2018).^28,29^ Each province was at a different stage of the scale-up process, providing a richer understanding of the various stages of implementation.^26,28^

###  Data Collection 

 Between August 2018 and April 2021, we collected data for each jurisdiction as well as data from national organizations (across provinces). Our data sources included non-participant observations, reviews of internal documents, and interviews (see Table 1). We continued data collection until we reached empirical data saturation within each jurisdiction.

**Table 1:** Data Collection for Each Jurisdiction

	**Methods**		
	**Non-participant Observation Meetings**	**Review of Internal Documents **	**Key Actor Interviews **
Ontario	19	35	11
Quebec	22	26	8
Manitoba	16	21	9
Newfoundland & Labrador	8	5	7
National	3	6	5
Total	68	93	40

####  Non-participant Observations

 We observed provincial eConsult steering committee meetings (n = 65) selected based on agenda items. Participants of the steering committee meetings included primary care professionals, specialists, policy-makers, researchers, information technology (IT) experts, and patients. These meetings focused on the spread and provincial scale-up of eConsult and helped us to better understand the contexts and gather monthly data on what was happening in each province. We collected data using an observation form we developed based on the key enabling factors for scale-up identified in the literature^1,4,15,30^ as well as elements deemed essential by knowledge users for the implementation of eConsult at a provincial scale. We completed the observation form, which has been previously published elsewhere,^25^ live during the meetings. Two PhD-trained research professionals (MAS and CLL) conducted initial observations to test the form and achieve consensus on its use. CLL conducted the remaining observations. In addition, we used the same form to observe three National eConsult Forums (2018 – in person, 2019 – in person, 2020 – virtual) where 75 to 150 provincial and national actors (eg, policy-makers, patient partners, family physicians, specialists, researchers, partner organizations) discussed scale-up strategies.

####  Internal document review

 We collected internal documents (n = 93), such as meeting minutes, PowerPoint presentation slide decks, working documents, policy documents, and scale-up plans, either directly from key actors or by observing committees.

####  Semi-structured interviews

 We conducted semi-structured interviews (n = 40) with key actors in each province (eg, researchers, primary care professionals, specialists, policy-makers, patient partners). To capture a variety of perspectives, we recruited participants with varied roles in the scale-up process and experience with and knowledge of eConsult. We recruited all key actors participating in provincial eConsult committees and those involved in eConsult implementation at the regional, provincial, and national levels. The researchers and knowledge users on our team helped us identify key actors to approach for participation. We adapted the interview guide from a previously published guide^25^ and tailored it according to each participant’s role and province. Two researchers with expertise in qualitative interviewing (MAS and CLL) who had not played a role in the scale-up of eConsult conducted the interviews to ensure confidentiality and minimize the influence of the interviewers on the answers provided. We digitally recorded and transcribed all interviews verbatim.

###  Data Analysis 

 We based our analysis on the main factors identified in the literature that enable the scale-up of health innovations. Our initial thematic analysis^31^ drew inspiration from Roger’s foundational work on the diffusion of innovations,^30^ the 20 scale-up success factors identified by Milat et al,^4^ factors identified in a literature review published by Ben Charif and al,^1^ and factors identified in a study on factors influencing the scale-up of eConsult published by Moroz and al.^15^ We imported transcripts of the 40 interviews (lasting 60 minutes on average) into NVivo 12. Observation notes informed our analysis. First, CLL and MAS coded four transcripts, discussing divergences in their codes after each one. Once they reached coding agreement, CLL coded the remaining transcripts. Second, based on the codebook, CLL wrote case narratives for each province detailing context, strategies (eg, events, decisions, and actions), and enabling factors and their influence on the scale-up process. Two other team members (MB and MAS) reviewed these narratives for clarity and completeness. Three researchers on the team (MB, MAS, and CLL) then analyzed strategies across cases, grouping similar strategies and categorizing them within the key enabling factors identified in the literature review. Finally, in May 2021, we held two 60-minute virtual meetings to share, discuss, and validate the key enabling factors and related strategies with team members from the four provinces who were either involved in scaling up eConsult or had expertise in scaling up innovations. We organized two meetings based on the same content to reach as many participants as possible. At these meetings, we used interactive methods (eg, chat box, virtual sticky notes, and live discussions) to facilitate a structured discussion around the trustworthiness and confirmability of the preliminary findings.

## Results

 We studied the process of scaling up eConsult in four provinces. Table 2 lists the characteristics of each of the jurisdictions.

**Table 2:** Jurisdiction Characteristics

	**Year eConsult Started**	**Volume of eConsults (2020)**	**Average Population Served/Million Inhabitants (2020)**	**Summary of eConsult Scale-Up Progression**
Ontario	2009	65 672	14.7	Province-wide scale-up began in 2018 Service managed through the Ontario eConsult Centre of Excellence, which was created in 2018 Provincial pro-rated fee for specialists (2009) and fee-for-service tariff for PCPs (2012)
Quebec	2017	2446	8.6	Pilot project in three regions ran from 2017 to 2021 Province-wide service launched in May 2021 Provincial fee-for-service tariff approved for specialists and PCPs (June 2021) Service integration with regional dispatch centers for referrals to specialists was underway as of August 2021
Manitoba	2017	1926	1.4	Pilot project ran from 2017 to 2021 SharePoint platform set up for pilot project used during the transition period (beginning April 2021) Specialists paid (pro-rated fee) through transitional funds from government Spread put on hold in April 2020 Onboarding, support, and training managed through provincial infrastructure as of April 2021
Newfoundland & Labrador	2016	3074	0.5	Developmental project ran from 2016 to 2020 Provincial service integrated into provincial EMR launched in 2020 Fee-for-service tariff approved for specialists in May 2020 Ongoing scale-up of the service province-wide

Abbreviations: EMR, electronic medical record; PCPs, primary care professionals.

**Figure 2:**
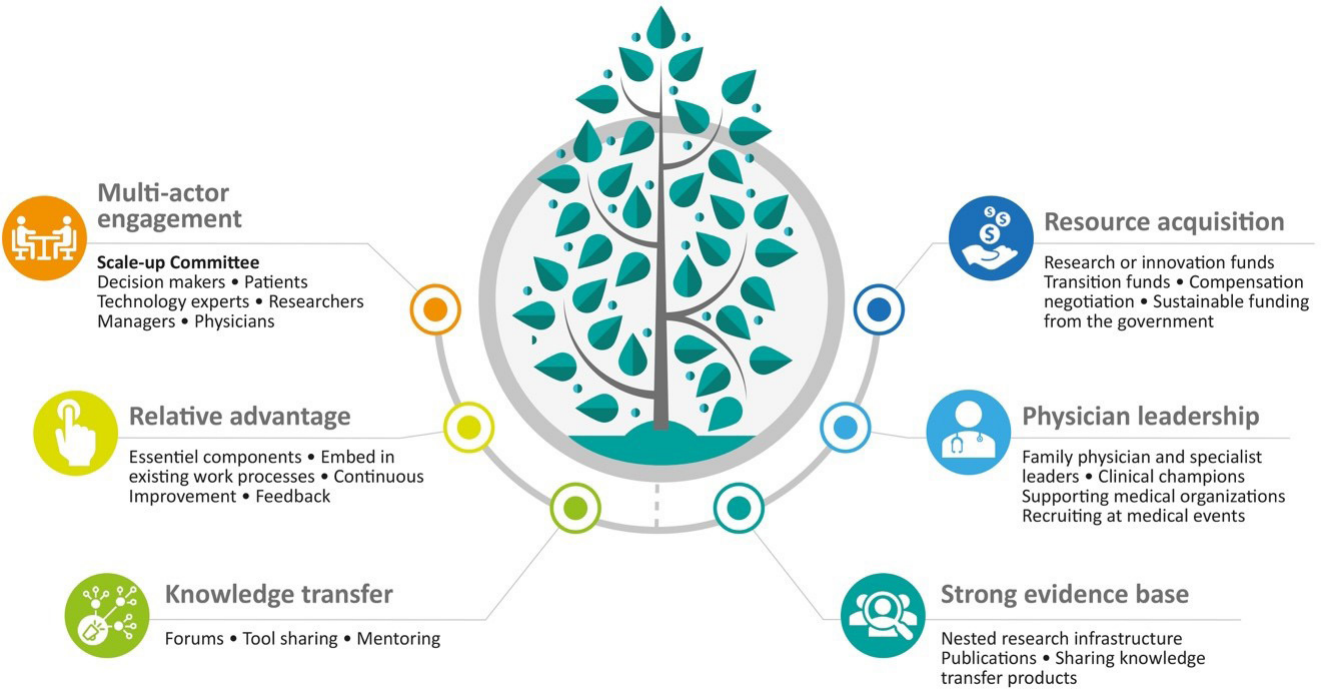
Summary of Key Factors Enabling eConsult Scale-Up and Strategies Used to Address Them.

 Based on case narratives and team discussions, we identified six common enabling factors across the four provinces, which we refer to as key factors enabling eConsult scale-up: (1) multi-actor engagement; (2) relative advantage; (3) knowledge transfer; (4) strong evidence base; (5) physician leadership; and (6) resource acquisition. Below, we present each factor in turn, explaining why it is key to the scale-up of eConsult and describing the strategies used to address it. Figure 2 summarizes the factors and strategies.

###  Multi-actor Engagement 

####  Why Is This Enabling Factor Key to the Scale-Up of eConsult? 

 Ongoing engagement of multiple key actors — including patient partners, policy-makers, primary care professionals, specialists, IT experts, and researchers — helped to strategically align the implementation of eConsult with provincial health system priorities and adapt to changing policy contexts. Additionally, engaging various actors from a broad range of organizations and with varied perspectives allowed for early identification of potential policy and infrastructure issues that needed to be addressed and helped to navigate complex policy processes.

####  What Strategies Were Used? 

 In each province, the engagement of key actors began with the physician leaders who first piloted the innovation. They recruited a team of knowledge users and patient partners through their personal and professional networks to prepare and apply for one or more research grants. Research funds facilitated the assembly of these teams, and collaborating on a concrete project in this way helped to build their interest and commitment. Motivated and action-oriented strategic actors received invitations to participate in research grants. As eConsult was spread and scaled up, steering committees enlisted additional members to provide insights into emerging issues and recruited specific high-level policy-makers to participate on the committee and act as representatives of their organizations (eg, ministries of health, medical associations). Provincial eConsult physician leaders chaired these meetings. Throughout the scale-up process, provincial eConsult steering committees that met once or twice per month maintained actor engagement for the most part. At these meetings, physician leaders and researchers provided updates on evaluation results (eg, eConsult volumes, wait times), members whose organizations were directly involved in the scale-up process updated the committee on their progress, and committee members discussed emerging challenges, brainstormed solutions, and advised high-level actors on strategic orientations. Beyond these meetings, the research team ensured ongoing engagement of key actors by providing communication tools they could use to advocate for eConsult (eg, presentation slide decks, policy briefs, and letter drafts). Moreover, they celebrated eConsult milestones and successes with all those involved to help build a sense of ownership among them. Furthermore, the Ontario team invited actors from around the country to participate in National eConsult Forums held in collaboration with the Royal College of Physicians and Surgeons of Canada. Participants perceived these forums as fostering further engagement, particularly in provinces that were at the earlier stages of scaling up eConsult. At these forums, 75–150 individuals discussed the potential value of scaling up eConsult. Inviting national-level policy-makers (eg, College of Family Physicians of Canada, Royal College of Physicians and Surgeons) to these forums provided them with an opportunity to discuss national issues, such as interjurisdictional licensing and inclusion of eConsult in medical curricula, to brainstorm solutions, and to stay up-to-date on the progress of scale-up in each province.

 Various actors played different complementary roles in the scale-up of eConsult. High-level actors (eg, policy-makers, representatives of medical associations, and physician leaders) participated as spokespeople who endorsed eConsult and advocated for its scale-up among influential actors. Patient partners encouraged the use of several strategies, such as evaluating eConsult from the patient perspective, acting as strong advocates for eConsult, drafting jargon-free promotional materials, and including rich patient stories to promote eConsult.

 “*I just think that the patient reps and the stories that they bring are the most important part of the development project because we need to be listening to them. We need to be learning from them*” (Newfoundland & Labrador).

 Multiple types of actors spread a similar message about the need to scale up eConsult through their respective communication channels, generating momentum and excitement across the country.

Table 3 presents a summary of the strategies used in each province to address multi-actor engagement.

**Table 3:** Strategies Used to Address Multi-actor Engagement

	**Ontario**	**Quebec**	**Manitoba**	**Newfoundland & Labrador**
Work with multiple types of actors, including knowledge users and patient partners, to prepare research grant applications	√	√	√	
Recruit actors strategically through personal and professional leaders’ networks	√	√	√	√
Create a steering committee to oversee eConsult pilot project and spread	√	√	√	√
Maintain engagement for the duration of the scale-up process (eg, follow-up regularly, provide real-time results, celebrate milestones and accomplishments)	√	√	√	
Provide ready-to-use communication materials actors could use to advocate for eConsult	√	√	√	√
Invite a variety of actors to participate in annual National eConsult Forums	√	√	√	√

###  Relative Advantage 

####  Why Is This Enabling Factor Key to the Scale-Up of eConsult? 

 Our results suggest that, in all provinces, a variety of key actors perceived eConsult to be highly useful and to have a strong relative advantage over the status quo or other innovations. This positive perception facilitated its widespread adoption by health service professionals and, hence, its scale-up.

####  What Strategies Were Used? 

 eConsult was designed to be as simple as possible for professionals to use. Several respondents highlighted the importance of developing a simple intervention that facilitated its implementation and use. Participants perceived eConsult as being easy for potential adopters and policy-makers to understand and for professionals to use in everyday practice. Furthermore, it was seen as being easy for organizations to manage changes required for its implementation. In particular, participants noted that being a simple, streamlined innovation that integrates well into professionals’ workflows set eConsult apart from other innovations.

 “*What makes it different is how it is embedded in the workflow. So, it’s convenient…” *(Manitoba).

 For example, respondents felt that the use of a standardized form with only four mandatory questions facilitated its integration into the workflows of primary care professionals. Although many specialists requested that the form be tailored to their specific needs, the Ontario team decided, based on their experience and user feedback, to use one standardized form with only four fields applicable to any medical specialty. They felt that this would be less burdensome for primary care professionals and, therefore, increase the likelihood of them using it. Our results show that this simplicity helped to avoid delays in scale-up because the design focused on core elements and limited “scope creep,” particularly in terms of IT developments and integration with electronic medical records. This idea of simplicity achieved consensus among the interviewees.

 “*…developing and scaling integrated things is much harder than developing and scaling dead simple things. So, the trade off, I think it’s easier to scale something dead simple*…” (Ontario).

 “*…The conclusion that I have made is that, even if it’s not advanced computing, [...] the technical solution has to be simple and user friendly and just easy to use and adopt” *(Manitoba).

 One of the main strategies for development, spread, and scale-up was to begin by addressing users’ needs and to subsequently adapt to their preferences while maintaining a functional eConsult service.

 To ensure a relative advantage in terms of usability and ease of implementation, and to reduce adoption burden on professionals, the design of eConsult mirrored traditional referral processes as much as possible. For example, mandatory close-out surveys (all provinces) collected user feedback, and a user advisory committee (Manitoba) adapted the service to user feedback (eg, problems, frustrations, and suggested improvements) to make it user-friendly and ensure integration into the existing workflows of specialists and primary care professionals. Research teams regularly analyzed responses and used them to continually improve the innovation.

 Refinement of the eConsult model largely took place during the stages of the pilot project in Ontario. The other provinces followed this model on the BASE^TM^ platform (Manitoba, Newfoundland and Labrador) or on a local platform managed by a private company (Quebec). In the three other provinces, changes made to eConsult related mainly to technical issues or requests to add new specialties to the service.

 In all provinces studied, regional coordinators provided training and ongoing support to help professionals with the onboarding process to the eConsult platform and to troubleshoot issues. In some cases, clinical administrative staff were trained or a train-the-trainer approach was used to ensure access to local support. Training videos and guides, remote virtual training sessions, and in-person training further facilitated adoption and implementation.

 Integrating eConsult with other existing services was another strategy used to ensure its relative advantage. Although the eConsult platform started as a standalone service, both Newfoundland & Labrador and Ontario integrated eConsult with existing services within electronic health records or with other telemedicine services. Newfoundland & Labrador’s use of a single electronic health record facilitated eConsult’s integration across the province. Ontario integrated eConsult into targeted electronic health records as a pilot project. In Quebec, in 2021, work was underway to integrate eConsult within regional dispatch centers for referrals to specialists.

 To ensure potential adopters were aware of eConsult’s relative advantages (eg, simplicity, effectiveness, ease of use for professionals, and usefulness for patient care), information about eConsult was promoted through a variety of channels, including institutional newsletters, social media, news media (eg, newspaper advertisements and press coverage), eConsult websites, information booths at relevant events, word of mouth (eg, physician champions and patient partners), webinars, presentations, policy briefs, infographics, and promotional material. In Ontario, traditional referral requests that could be answered by eConsult were often returned to the primary care professionals with a recommendation to send the request as an eConsult, thereby helping to promote the service. Promotional messages describing eConsult and promoting its relative advantages were evidence-based, focused, and consistent. For example, Canada Health Infoway created short videos featuring professionals’ testimonials about their positive experiences with eConsult for publication on their website. Patients’ stories of the perceived benefits of eConsult also promoted eConsult to policy-makers. Finally, the favouring of images over words (eg, infographics and images) increased the accessibility and retention of the promotional messages.

Table 4 presents a summary of the strategies used in each province to address relative advantage.

**Table 4:** Strategies Used to Address Relative Advantage

	**Ontario**	**Quebec**	**Manitoba**	**Newfoundland & Labrador**
Maintain simplicity of the eConsult design to focus on its core elements	√	√	√	√
Ensure eConsult is user-friendly and can be integrated into users’ workflows	√	√	√	√
Offer training and ongoing support to encourage uptake and troubleshoot issues	√	√	√	√
Integrate eConsult into electronic health records and other eReferral services	√	√		√
Promote the relative advantages of eConsult (eg, healthcare organization newsletters, eConsult websites, word of mouth, webinars, fax backs sent by specialists to primary care professionals, and patient stories)	√	√	√	√

###  Knowledge Transfer 

####  Why Is This Enabling Factor Key to the Scale-Up of eConsult? 

 Transferring learnings from Ontario to the other provinces helped to accelerate some aspects of the scale-up process by enabling them to benefit from other’s experiences. Additionally, it generated excitement about eConsult among key actors and increased national momentum for its spread and scale-up. Finally, this key enabling factor created positive peer pressure among provincial actors.

####  What Strategies Were Used? 

 Ontario was the first province to begin scaling up eConsult, and they freely shared their evidence and tools (eg, business case, policy brief, implementation guide, training tools; see https://www.champlainbaseeconsult.com/) with the other three provinces. For instance, Ontario leaders shared the eConsult platform they developed with other jurisdictions for a small system maintenance fee. Ontario leaders also shared lessons learned, experiences acquired from spreading and scaling up eConsult, tools, and strategies used to address the challenges they encountered. In doing so, the provinces were able to benefit from the work others had done and adapt it to their jurisdiction, saving them the time and effort of starting from scratch. As a result, they drafted plans to avoid some of the challenges others had faced and devised creative solutions to overcome known barriers to scaling up eConsult. The National eConsult Forums also played a fundamental role in supporting knowledge transfer. At each edition of the forum, participants not only shared learnings from their jurisdiction but also collectively brainstormed potential solutions to challenges.

 “*I think one of the first turning points for me was the national gatherings – the forum. There were a lot of ‘ ahas ’ in the room. People were curious. [...] The forum is a great opportunity to showcase success, discuss challenges but also to sell the concept.* […] *For those who are less sure about it, to be in a room, to talk about it for a day and a bit, it’s difficult to walk away not thinking that this is a good idea” *(National).

 eConsult was part of the CFHI’s Connected Medicine Collaborative, which engaged key actors from different provinces in a formal program aiming to help spread remote consultation services (eg, eConsult and phone consultations). The work of the CFHI collaborative included hosting webinars, developing business cases, establishing governance and scale-up plans, providing coaching from innovation leads, and organizing regular meetings to foster the sharing of lessons learned across provinces.

 “*I think it was really good to be involved from the get-go with the other folks that are doing similar things across the country. Having that opportunity to collaborate and to meet in person, in particular to learn what others are doing, was very valuable. It helped us to figure out what kind of metrics we should be looking at from the get-go” *(Newfoundland & Labrador).

 Ontario’s eConsult leads also acted as mentors to those in other provinces. They regularly participated in eConsult steering committee meetings and in discussions with high-level policy-makers by presenting evidence and offering suggestions. This enabled the leads to remain informed of progress in each jurisdiction and facilitated the exchange of strategies used among jurisdictions. For instance, they shared insight into various strategies to build a convincing rationale for scaling up eConsult and for securing funds from alternative sources to help transition eConsult from a research project to a sustainable innovation.

Table 5 presents a summary of the strategies used to address knowledge transfer.

**Table 5:** Strategies Used to Address Knowledge Transfer

	**Ontario**	**Quebec**	**Manitoba**	**Newfoundland & Labrador**
Share evidence, experience, knowledge, and tools (either free-of-charge or for a nominal fee)	√			
Hold National eConsult Forums to facilitate shared learning and collectively brainstorm potential solutions to challenges	√			
Mentoring from pioneers through the CFHI Connected Medicine Collaborative	√			
Provide active mentoring to actors in other provinces by participating in provincial steering committee meetings	√			

Abbreviation: CFHI, Canadian Foundation for Health Improvement.

###  Strong Evidence Base 

####  Why Is This Enabling Factor Key to the Scale-Up of eConsult? 

 Research funds initially supported the development and piloting of eConsult, and research played an important role throughout all stages of eConsult’s development. The fact that eConsult was embedded within a research infrastructure fostered the creation of a strong evidence base. Several domains contributed data to the evidence base, including the effectiveness (number of non-urgent in-person visits avoided, patient satisfaction, increased access to specialized care), cost-effectiveness (cost per eConsult, cost per eConsult avoided), feasibility (growth projections for volume of eConsults), acceptability (primary care professionals and specialist satisfaction), adoption (volume per professional, number of active professionals), and potential reach (number of eConsults completed per clinic and region) of the eConsult pilot projects. This large evidence base, built over 12 years, lent credibility to the innovation and demonstrated the benefits and value of eConsult to policy-level actors and funding organizations.

 “*I feel that the publications that Clare and Erin (co-founders) have done over the years add credibility and provide a lot of expertise into informing the model. […] I think it’s instrumental to informing the policy decisions and I think it was a key success factor in making the case for eConsult to be a provincial program*” (Ontario).

 The well-established evidence base also helped address actors’ concerns regarding patient safety and acceptance of the service. For instance, actors initially questioned the safety of an eConsult compared to an in-person appointment. However, the evidence indicating that only a small proportion of eConsults led to in-person appointments with a specialist, despite this not having been initially planned by the primary care professionals, reassured them. Additionally, in each province, the current evidence base generated by the research team informed policy discussions and negotiations. For example, economic evidence from Ontario on the cost per eConsult and volume data informed remuneration negotiations in other provinces. Needs assessments also helped eConsult teams establish spread and scale-up priorities, such as implementing eConsult for specialties with long wait times or in areas where access to specialists was limited.

####  What Strategies Were Used? 

 Our results suggest that embedding eConsult within a research infrastructure helped to create a continuously-growing evidence base. Beginning with the pilot studies in the provinces, a research infrastructure was created and maintained throughout the spread and scale-up of eConsult. Research grants obtained from local, provincial, and national funding bodies financed the human resources needed to implement the eConsult service and maintain it until funding was institutionalized, covered fees for specialists’ time, and allowed for evaluations and needs assessments to be conducted to explore further implementation (eg, in correctional and long-term care facilities) and uses (eg, continuing medical education). Furthermore, negotiations with provincial health authorities regarding long-term funds for sustainability resulted in a portion of the budget being dedicated to the continuing evaluation of the service.

 The collection and analysis of relevant data (eg, audits, mandatory close-out survey, and patient and professional surveys to assess effectiveness, cost-effectiveness, feasibility, acceptability, adoption, and potential reach) using quantitative and qualitative research methods built the evidence base. A variety of knowledge translation strategies shared the results of the research projects and the evidence base to inform scale-up. Over 100 scientific publications were produced, primarily by the Ontario team, providing a credible, peer-reviewed body of literature to build a convincing rationale to scale-up eConsult. Other provinces perceived the data collected in Ontario as being transferable to their own contexts.

 “*I think [the evidence base] really informed and shaped how they (the co-founders) do deployment, how they frame the benefits of the system... It’s much easier, I think, for people to go to a doctor’s office and say, ‘You should use this system. It’s really valuable,’ when they have evidence to back that up*” (Ontario).

Table 6 presents a summary of the strategies used in each province to address a strong evidence base.

**Table 6:** Strategies Used to Address a Strong Evidence Base

	**Ontario**	**Quebec**	**Manitoba**	**Newfoundland & Labrador**
Embed eConsult within research infrastructure (research grants to support the innovation)	√	√	√	√
Negotiate provincial funding to continually evaluate the service	√			
Use quantitative and qualitative research methods to evaluate the innovation (eg, think tank discussions, interviews, surveys, audits)	√	√	√	√
Embed research into quality improvement efforts	√	√	√	√

###  Physician Leadership 

####  Why Is This Enabling Factor Key to the Scale-Up of eConsult? 

 Two physician leaders in Ontario, one family physician and one specialist, pioneered the initial development and piloting of eConsult. We defined a physician leader as a physician who played a key role in deploying the intervention. Originally, eConsult emerged from clinical needs and was adapted to the reality and workflow of professionals (ie, innovation users). In addition, the engagement of physician leaders was a key factor in identifying priorities for expanding eConsult to other specialties. For example, in all provinces studied, primary care professionals determined the order in which the eConsult service integrated specialties based on their observations and experiences regarding patient needs as well as specialist care services with the longest wait times. Strong physician leadership also helped to gain valuable support from provincial and federal medical associations for scale-up and to secure their participation in the process. Physician leaders recruited local eConsult users as clinical champions to promote the innovation among their colleagues. This facilitated the recruitment of additional family physicians and specialist users and lent credibility to the innovation, thereby increasing its uptake and adoption among professionals.

####  What Strategies Were Used? 

 Ontario and Quebec formalized the role of physician leader in the eConsult scale-up process. Specifically, the Ontario eConsult Center of Excellence named the two initial physician leaders as co-executive directors, and Quebec’s Ministry of Health and Social Services appointed the main physician leader in Quebec as a medical advisor on eConsult. Manitoba and Newfoundland & Labrador used this strategy of formalizing the role of physician leaders less often.

 The CFHI’s Connected Medicine Collaborative engaged several physician leaders in each province, providing training, webinars, and tools designed to encourage and support clinicians to take on key roles in the spread and scale-up of eConsult in their province.

 During the eConsult pilot projects in each province, physician leaders used their personal networks in a strategic way to build a clinical “coalition of the willing” (ie, enthusiastic professionals who were convinced of the benefits of the innovation) to advocate for eConsult among their colleagues. This provided momentum to increase the use of eConsult and recruit additional professionals to join the coalition.

 Physician leaders recruited local physician champions (ie, clinicians who believed in the innovation and were willing to help recruit their local colleagues) as volunteers from among the attendees of their eConsult presentations at scientific and medical conferences (ie, Family Medicine Forum, North American Primary Care Research Group) and at regional tables. Physician champions shared their eConsult experience with their peers, encouraged them to become users, and acted as a resource to help resolve minor issues that users faced. They did not receive incentives for their role as champions.

 Physician leaders also sought the endorsement of provincial and federal medical associations to help grow the provincial and national momentum surrounding eConsult and provide support for scale-up efforts (eg, funding, negotiations, political influence, and promoting eConsult to members).

 “*I think [the medical associations’] biggest role in the collaborative was helping get the word out and putting their stamp of approval. Like saying this is something that we can get behind even before they put it as a standard of practice” *(National).

Table 7 presents a summary of the strategies used in each province to address physician leadership.

**Table 7:** Strategies Used to Address Physician Leadership

	**Ontario**	**Quebec**	**Manitoba**	**Newfoundland & Labrador**
Formalize the role of physician leaders	√	√		
Train and mentor physician leaders through a national collaborative	√	√	√	√
Form a coalition of enthusiastic professionals from the outset	√	√	√	√
Engage physician champions through scientific and medical conferences and regional tables	√	√	√	
Seek endorsement from provincial and federal medical associations to grow momentum and support scale-up efforts	√	√	√	√

###  Resource Acquisition 

####  Why Is This Enabling Factor Key to the Scale-Up of eConsult? 

 We identified the final key enabling factor as resource acquisition, which include human and financial resources to coordinate and manage eConsult, an adequate electronic platform, remuneration for primary care professionals and specialists, and continuous evaluation of eConsult. Our results suggest that it is essential to secure resources for three phases: (1) development and pilot testing; (2) transitioning from pilot to scale-up (to maintain achievements and spread eConsult to new contexts while policy issues are being addressed); and (3) ensuring sustainability (eg, recurring resources, including government funding, physician remuneration, and human resources to manage and oversee eConsult, as necessary, at the health system level).

####  What Strategies Were Used? 

 Since 2009, the eConsult team leads have applied for many research grants and innovation funds from various local, provincial, and national funding bodies (eg, Canadian Institutes for Health Research) to pilot and evaluate eConsult in various settings (eg, interdisciplinary chronic pain care and long-term care) and with various actors (eg, allied health professionals). Physician leaders, in close collaboration with professionals, patient partners, policy-makers, and researchers, wrote detailed study protocols included in grant applications. The CFHI’s Connected Medicine Collaborative provided key multi-jurisdictional funding to support pilot projects and to spread eConsult to new regions across different provinces.

 “*[The CFHI] really provided the infrastructure and leadership and funding for people to join the connected medicine collaborative, which has meant to really increase capacity across the country as well as implementation*” (Ontario).

 Our findings suggest that obtaining transition resources is important for maintaining achievements and spreading eConsult to new contexts while policy issues are being addressed. The transition period begins when initial research and innovation funds run out and teams must be creative to find sources of funding. However, obtaining transition resources can be difficult, as traditional Canadian funding bodies do not typically fund transition projects. In Quebec, Manitoba, and Ontario, eConsult teams and their partners advocated for eConsult to their respective provincial governments and obtained temporary transition funds to pay for specialists’ time (pro-rated fee used in research projects) and coordinate and manage the service when the research grant ended. This ensured the maintenance of eConsult services for onboarding professionals and for growth through a limited amount of spread until the establishment of fee-for-service tariffs. Non-traditional funding sources, including small funds obtained through innovation challenges or contests launched by the CFHI, medical associations, and family medicine departments, as well as in-kind resources from key partners (eg, regional organizations, research infrastructure, and medical associations) also supported the service until it was institutionally funded.

 Establishing fee-for-service tariffs or alternate funding for specialists and primary care professionals secured, for the most part, sustainable resources. In Ontario, the Ministry of Health and Long-Term Care funded the remuneration of specialists through a prorated fee of $200 CAD/hour. This rate was used in pilot projects in Ontario (beginning in 2009) and in the other three provinces for specialists paid through a fee-for-service payment model. Newfoundland & Labrador and Quebec approved fee-for-service tariffs for specialists in May 2020 and June 2021, respectively. Ontario and Quebec also secured provincial remuneration for primary care professionals from provincial payers. Members of the provincial steering committees and eConsult advocates participated in remuneration negotiations, leveraging the eConsult evidence base. Additionally, to advocate for the establishment of provincial physician remuneration, eConsult steering committee members used a combination of complementary strategies, including sending letters to the Minister of Health, requesting a fee code from provincial payers, and encouraging professionals to contact their medical association and request a tariff. New or existing provincial infrastructure also secured sustainable human resources. For instance, in Ontario the newly created Ontario eConsult Centre of Excellence took on the management of the eConsult service, including physician remuneration. In Newfoundland & Labrador, the existing provincial payer managed physician remuneration and monitored the number of eConsults per physician to ensure that previously established limits per professional (400 eConsults per year) were respected.

Table 8 presents a summary of the strategies used in each province to address resources for scale-up.

**Table 8:** Strategies Used to Address Resource Acquisition for Scale-Up

	**Ontario**	**Quebec**	**Manitoba**	**Newfoundland & Labrador**
Resources for development and pilot testing				
Secure initial funding through research grants and innovation funds	√	√	√	√
Resources for transition				
Advocate for eConsult to provincial governments to obtain transition funding to maintain eConsult services	√	√	√	√
Obtain funds from non-traditional sources to manage and coordinate eConsult during the transition period (eg, innovation challenges and contests, remaining research funds, in-kind resources from key partners, research infrastructure, medical associations)	√		√	√
Resources for sustainability				
Establish fee-for-service tariffs or alternate funding to secure sustainable funding	√	√		√
Enlist key actors to participate and advocate for eConsult in remuneration negotiations	√	√	√	√
Use complementary methods to advocate for the establishment of provincial physician remuneration (eg, sending letters to the Minister of Health, requesting fee codes from provincial payers, encouraging physicians to request a tariff from their medical association)	√		√	
Use existing and new infrastructure to provide human resources to sustain the service	√	√	√	√

## Discussion

 This study provides an in-depth analysis of the strategies used to scale up a promising innovation and related key factors based on data from four rich cases. For each key enabling factor, we described its importance to the scale-up of eConsult and identified and described the strategies used to address it. To our knowledge, our study is the first to distinguish between factors and strategies. By delving into the strategies used to address enabling factors and providing guidance on how to implement them, our paper makes a substantial and original contribution to the science of scale-up. Furthermore, as our study is one of the first conducted in a high-income primary care context like Canada,^4^ our results are unique within the literature.

 In this study, we identified a total of 31 strategies related to six key factors that enabled the scale-up of eConsult in four Canadian provinces: (1) multi-actor engagement; (2) relative advantage; (3) knowledge transfer; (4) physical leadership; (5) a strong evidence base; and (6) resource acquisition. These six identified factors are similar to those identified in the literature,^1,4,9,10,15,16^ although the labels and categories vary among articles.

 Similar to other studies,^1,15^ our results show that a key enabling factor is the engagement of a range of actors. We add to this by identifying six strategies that address this factor. These strategies include creating multi-actor steering committees to oversee eConsult pilot projects and spread and providing actors with ready-to-use communication materials they can use to advocate for eConsult.

 Another key factor identified as enabling the scale-up of eConsult was transferring learnings from Ontario to the other provinces. Four strategies addressed this factor, including sharing evidence, experience, knowledge, and tools, holding National eConsult Forums to facilitate shared learning, and mentoring other provinces. A previous study on key policy strategies for the spread and scale-up of eConsult highlighted the importance of building on current strategies and existing policies in the context of eHealth.^15^ Although that study did not report on shared learning, the authors proposed similar strategies to those we identified as enabling this factor in the present study.

 We identified physician leadership as a key enabling factor that was addressed by strategies such as formalizing the role of physician leaders, engaging physician champions, and seeking the endorsement of medical associations to grow momentum and support scale-up efforts. Similarly, Moroz et al^15^ highlighted the importance of the support provided by “change” champions.

 Additionally, we further refined the key enabling factor of evaluation and monitoring, which Milat et al^4^ found to be important, by suggesting that a strong evidence base is needed to enable scale-up and that this can be developed by embedding the innovation in research.

 We found that resource acquisition was a key enabling factor addressed by different strategies during the pilot and testing, transition, and sustainability phases. A recent study identified different funding models to move beyond initial financial resources.^32^ Long-term funding was reported as a key factor to enable sustainability. The authors proposed to include long-term fundings in proposals or plan agreements for funding renewal under certain conditions of innovation performance.^32^ In addition, the existing literature highlights commonly reported scale-up barriers and facilitators related to human, financial, and technical resources, the capacity and will to scale-up, relational and political connections, active engagement of diverse actors and organizations, leadership, approaches tailored to context, and evaluation.^1,3,4,9,10,33-39^

 Identifying enabling factors at play in different contexts is useful to understand how they influence the scale-up of innovations. However, knowing the necessary enabling factors is not sufficient. A better understanding of these strategies, including concrete actions that actors can take to support scale-up efforts and address key enabling factors, is crucial to advance our ability to scale-up promising health innovations. The strategies and factors we have identified and described are grounded in both empirical evidence and theoretical frameworks on the scaling up of innovations^1,4,30^ as well as key enabling factors identified in the literature.^15^ To our knowledge, this study is one of the first to provide ideas for actions that different types of actors with varying roles (eg, professionals, researchers, and policy-makers) and at multiple levels of governance (eg, regional, provincial, national) can take to scale up an innovation.

 In this research, we studied the scale-up of an innovation with demonstrated effectiveness that was expanded at the healthcare system level in four jurisdictions, each with its own health system. Dedicated teams implemented and supported many strategies, but they also described these strategies as very time and resource intensive. Many actors noted that the scale-up of innovations remains incredibly complex and is a constant “uphill battle” that is poorly supported by our health systems. Promising innovations need support to improve health systems through scale-up. Creating supportive infrastructures and programs may help innovators overcome the challenges of scaling up innovations.^40^ For instance, creating more national infrastructures that promote learning, increasing funding, and facilitating structured interactions between multiple actors across jurisdictions may enable scale-up of other innovations. To achieve this, one potential approach could involve the creation of “scale-up accelerators” that catalyze the process of aligning innovations with health system priorities, facilitate connections with relevant actors, provide scale-up coaching (eg, business case, scale-up plan), and help innovators navigate policy (eg, IT requirements, legislation, and resources).^20^ Laur et al^41^ proposed to support innovators and transition innovations from pilot projects to larger scale-up by creating capacity-building programs. These programs could facilitate the development of strong and long-term partnerships between key actors.^41^

###  Strengths and Limitations 

 In terms of limitations, it is worth noting that we may not have captured all of the relevant strategies employed in the provinces, particularly strategies for securing sustainable resources, as some of the provinces had not completed the scale-up process during the study period. Certain provinces started the scale-up process earlier, which might have influenced strategies used in other provinces. Furthermore, given that Canada provides universal healthcare, with distinct publicly-funded health systems in each province, the results observed in this study may not necessarily be replicable in other contexts. It is necessary to take contextual factors such as these into consideration when adapting these findings to other settings.

 We undertook several strategies to ensure the trustworthiness of our findings. The study design, which covered multiple contexts and involved the triangulation of data collected over 3 years, supports the credibility of our results, as does the peer debriefing conducted with all co-authors during two 60-minute virtual meetings. Moreover, we ensured confirmability by, for example, involving multiple co-authors in the data analysis, as described above.

## Conclusion

 The key strategies for scaling up an innovation that we identified and described offer concrete ideas for action that may help policy-makers, researchers, patients, and professionals who are interested in scaling up promising innovations. Scaling up innovations is context dependent, and our study provides strategies used in four jurisdictions in a high-income country to scale up an innovation. Further studies are needed to build an evidence base for key strategies that will help those who wish to scale up innovations while making efficient use of resources. Moreover, identifying and describing strategies that fail to address scale-up factors and why may contribute to understanding the complexity of scaling up innovations and how to ensure their sustainability. Building a comprehensive scale-up knowledge base will help actors bring their promising innovations to the masses for the benefit of all.

## Acknowledgements

 The authors would like to acknowledge the contribution of all stakeholders from the four provinces and thank them for accepting to collaborate on the study. We also wish to thank the Canadian Institutes of Health Research, the McGill Observatory on Health and Social Services Reforms and all organizations that provided matching funds and in-kind support.

## Ethical issues

 All participants gave written consent to participate in the study. This study was approved by the St. Mary’s Hospital Center Research Ethics Committee (Quebec, #SMHC-18-15), the Bruyère Continuing Care Research Ethics Board (Ontario, #M16-19-006), the Newfoundland & Labrador Health Research Ethics Board (NL, #2019.070), and the Bannatyne Campus Health Research Ethics Board (Manitoba, #H2019:307).

## Competing interests

 Clare Liddy and Erin Keely are co–executive directors of the Ontario eConsult Centre of Excellence, funded by the Ontario Ministry of Health. They co-founded the Champlain BASE^TM^ (Building Access to Specialists through eConsultation) eConsult service but do not retain any proprietary rights. Erin Keely answers eConsults through the service, fewer than one per month.

## Funding

 This study was funded by the Canadian Institutes of Health Research (#402867), Réseau-1 Québec, Fonds de recherche Québec-Santé, Research Centre - Charles-Le Moyne-Saguenay-Lac-St-Jean sur les innovations en santé, Initiative Patient Partenaire from the University of Sherbrooke, the Manitoba SPOR Primary and Integrated Health Care Innovation Network, C.T. Lamont Primary Health Care Research Centre, and the McGill Observatory on Health and Social Services Reforms. The funders played no role in the design of the study, collection, analysis, or interpretation of the data, or writing of the manuscript.

## Disclosure of Relationships and Activities

 Research grants from the Canadian Institutes of Health Research [grant number #402867], Réseau-1 Québec, Fonds de recherche Québec-Santé, Research Centre - Charles-Le Moyne-Saguenay-Lac-St-Jean sur les innovations en santé, Initiative Patient Partenaire from the University of Sherbrooke, the Manitoba SPOR Primary and Integrated Health Care Innovation Network, C.T. Lamont Primary Health Care Research Centre, and the McGill Observatory on Health and Social Services Reforms were received to support this work.
